# The Design, Usability, and Feasibility of a Family-Focused Diabetes Self-Care Support mHealth Intervention for Diverse, Low-Income Adults with Type 2 Diabetes

**DOI:** 10.1155/2016/7586385

**Published:** 2016-11-07

**Authors:** Lindsay Satterwhite Mayberry, Cynthia A. Berg, Kryseana J. Harper, Chandra Y. Osborn

**Affiliations:** ^1^Department of Medicine, Vanderbilt University Medical Center, Nashville, TN, USA; ^2^Center for Health Behavior and Health Education, Vanderbilt University Medical Center, Nashville, TN, USA; ^3^Department of Psychology, University of Utah, Salt Lake City, UT, USA; ^4^Department of Biomedical Informatics, Vanderbilt University Medical Center, Nashville, TN, USA

## Abstract

Family members' helpful and harmful actions affect adherence to self-care and glycemic control among adults with type 2 diabetes (T2D) and low socioeconomic status. Few family interventions for adults with T2D address harmful actions or use text messages to reach family members. Through user-centered design and iterative usability/feasibility testing, we developed a mHealth intervention for disadvantaged adults with T2D called FAMS. FAMS delivers phone coaching to set self-care goals and improve patient participant's (PP) ability to identify and address family actions that support/impede self-care. PPs receive text message support and can choose to invite a support person (SP) to receive text messages. We recruited 19 adults with T2D from three Federally Qualified Health Centers to use FAMS for two weeks and complete a feedback interview. Coach-reported data captured coaching success, technical data captured user engagement, and PP/SP interviews captured the FAMS experience. PPs were predominantly African American, 83% had incomes <$35,000, and 26% were married. Most SPs (*n* = 7) were spouses/partners or adult children. PPs reported FAMS increased self-care and both PPs and SPs reported FAMS improved support for and communication about diabetes. FAMS is usable and feasible and appears to help patients manage self-care support, although some PPs may not have a SP.

## 1. Introduction

Family members and other close loved ones participate in patients' daily routines and are often present for self-care activities (e.g., food selection, meal preparation, and disease-related problem solving and coping) [1–5]. For adults with type 2 diabetes (T2D), the receipt of helpful actions (i.e., instrumental support) is more predictive of adherence to self-care than other types of support (e.g., emotional support) [2, 6, 7]. According to both social control theory [8, 9] and family systems theory [10, 11], family members are well-positioned to provide instrumental support for diabetes self-care and to create contexts valuing and supporting self-care adherence. However, patients who experience more harmful actions (e.g., nagging, arguing, and sabotaging self-care efforts) are less adherent to self-care [2, 12, 13] and have worse glycemic control [12]. Moreover, helpful and harmful actions are each strongly associated with patients' being more or less adherent to diet and exercise recommendations, respectively [12].

According to social control theory, certain actions are harmful because they are misaligned with the types of support patients need [8, 14] and/or infringe on their autonomy and create resentment [15] (e.g., nagging, arguing about self-care). According to family systems theory, harmful actions undermine patients' efforts to initiate and sustain self-care efforts (i.e., undermining or sabotaging self-care efforts, such as bringing tempting unhealthy foods into patients' homes) [10, 11]. Families who become involved in adults' self-care perform both helpful and harmful actions [2, 12, 16–18], but family interventions for adults with T2D have not adequately addressed the harmful aspects of family involvement [19].

Family interventions targeting both helpful and harmful actions may be particularly useful for racial/ethnic minorities and persons with low socioeconomic status (SES) with T2D. These groups have high rates of limited health literacy [20], more life stressors [21–23], and depression [24, 25], which may make them more vulnerable to the detrimental effects of others' harmful actions on their diabetes self-care and control [12, 26]. Family interventions may also be challenging for patients with T2D who (a) have diverse living and family situations [1, 27, 28], (b) live apart from the person(s) providing the most support [27, 28], (c) have difficulty attending and/or bringing family members to in-person interventions due to competing priorities and limited resources [4, 27, 29], and (d) are concerned family involvement will undermine their autonomy and self-efficacy [27]. On the contrary, family members want to be more involved in adults' diabetes management and often feel frustrated when they do not know how to best help [30]. Thus, patients may appreciate and benefit from one-on-one guidance on how to identify and communicate their desires and needs for specific supportive actions from family members [30].

In other chronic disease contexts (e.g., cardiovascular diseases, cancer, and arthritis), family interventions have significant and stable effects on health over and above patient-only interventions [31] but have been less successful in T2D [19, 32]. Therefore, we developed FAMS (Family-Focused Add-On for Motivating Self-Care) to help patients identify a diet or exercise goal, get desired support from family members or close loved ones, and redirect or cope with undesired or harmful actions without compromising their own health goals. FAMS seeks to improve patients' adherence to diet and exercise recommendations via increased self-efficacy, increased receipt of helpful actions, and reduced receipt of harmful actions.

FAMS is delivered via basic mobile phone technology (i.e., phone calls and text messages), which is ubiquitously available in the USA [33], even among adults with the lowest SES and racial/ethnic minority groups [34, 35]. A robust, multistep approach is recommended to develop effective mHealth interventions for patients with diabetes, particularly for underserved or vulnerable patients [36]. Therefore, FAMS was developed from (a) front-end qualitative and quantitative formative research with adults with T2D [2] and low SES [12, 27], alongside (b) early feedback from potential users (adults with T2D and low SES) and (c) members of our research team (who used and critiqued FAMS during internal beta testing), followed by (d) iterative usability and feasibility testing with potential users. Our objectives were to develop a family-focused intervention acceptable to patients receiving care from Federally Qualified Health Centers (FQHCs), obtain feedback and data to improve the intervention, and ensure our research processes were sound prior to an evaluative trial.

## 2. User-Centered Intervention Design

For mHealth interventions in diabetes [36, 37], user-centered design entails formative research prior to and during intervention development to understand the needs, values, and abilities of users, as well as iteratively assessing the design to improve users' perceptions of and interactions with the technology and content. Following these principles, we developed FAMS to harness universal text messaging (i.e., not requiring a smartphone or the Internet). To accommodate the diversity of family types among adults with T2D [1, 27, 28, 38], we designed FAMS to acknowledge that patients' “families” include whomever the patient considers included, regardless of legal/biological relationships. FAMS content is sensitive and applicable to various living arrangements (e.g., living alone, with children, or with friends/roommates). FAMS text messages were designed to be ≤6th-grade reading level and tested with the Flesh-Kincaid Grade Level and Automated Readability Index (tested with https://readability-score.com/). To ensure plain language and accommodate those with literacy limitations, we ensured each sentence expressed one thought, simplified sentence structure, and used active voice [39]. We then replaced or plainly defined multisyllabic (≥three syllables) and uncommon words and medical jargon [39]. Messages avoid references to potentially unavailable resources (e.g., gym memberships). We worked with a digital content developer to shorten messages to ≤160 characters (a common limit for text messages) while maintaining their meaning.

### 2.1. FAMS Intervention Components

Each FAMS component is briefly described below and in Table 1 with example messages. Phone coaching seeks to improve the patient's ability to identify family members' actions that support or impede his/her self-care goals and his/her skills and self-efficacy to ask for needed support and manage harmful actions to meet these goals. Authors LSM and KJH, who have graduate degrees in counseling, developed FAMS phone coaching to be deliverable by counselors/counselors-in-training or health coaches/health coaches-in-training (i.e., persons with training in helping skills). FAMS coaching occurs with the PP* alone* and combines family therapy with basic health coaching. Among adults with T2D, health coaching has successfully improved adherence to exercise recommendations [40], psychological functioning [41], and illness-related coping [41]. FAMS coaching employs evidence-based techniques from goal setting theory [42] (assessing current behaviors and problem solving and assessing confidence to achieve a goal and revising goals with a confidence rating <7 on a 10-point scale), cognitive behavioral therapy [43, 44] (role playing, homework), and best practices in health communication [45] (teach back). FAMS sends the PP one-way and two-way/interactive text message support tailored to his/her self-care goal selected during coaching and preferred time of day (Table 1).

Patients can* choose* to invite a support person (SP) regardless of relationship type or living arrangement to receive text messages (Table 1). PPs were encouraged to select someone who is part of their daily life and routine and not someone with whom they “have a lot of conflict.” The SP does* not* have to be the identified family member in the coaching session. An enrolled SP receives messages tailored to the PP's name, gender, and goal type (Table 1). These aim to help SPs be thoughtful about the support they provide and to increase communication about diabetes and the PP's goal. The SP text messages do not provide information on the PP's goal achievement to avoid encouraging nagging/arguing.

### 2.2. Community Engaged Research Studio

Before developing FAMS functionality, we shared FAMS design and content with adults with T2D through a Community Engaged Research Studio (CES) [46] and made improvements based on their recommendations. The CES, conducted through the Meharry-Vanderbilt Community Engaged Research Core, employs direct feedback from community members (“experts”) who share similar demographic characteristics of a researcher's desired sample to identify and address concerns (e.g., cultural appropriateness of study materials, fair compensation, and intervention/survey design) [46]. Our CES experts (~12) were diagnosed with T2D, predominantly African American (AA), and with low SES - no family members were included. The CES facilitator read each text message and participants shared feedback ranging from “good, like it” to discussions with multiple view-points expressed. Authors LSM and KJH asked follow-up or clarifying questions as needed during the CES; afterwards, the facilitators compiled and provided notes.

CES experts provided helpful feedback. They did not like messages presuming patients were struggling to meet their goal or that evoked strong negative language. For example, in one text message they advised the word “dangerous” was too negative: “Traveling is dangerous for diet goals.” They recommended it be changed to the following: “Traveling can cause you not to meet your diet goals.” CES experts also recommended defining “self-care” to avoid confusion, so we replaced this phrase with a specific self-care behavior (e.g., diet or exercise) or “taking care of your diabetes.” Community experts said they would feel comfortable inviting a SP and provided feedback on text frequency (recommended daily) and coaching frequency (most recommended every two weeks or monthly) and duration (most recommended 20–30 minutes). Finally, experts were enthusiastic about community clinic recruitment.

## 3. Iterative Feasibility and Usability Testing

FAMS functionality was developed and tested for usability alongside REACH (Rapid Education/Encouragement And Communications for Health), a text messaging intervention supporting diabetes self-care [47]. REACH (a) sends tailored messages addressing users' barriers to diabetes medication adherence, (b) sends nontailored messages addressing adherence to diet, exercise, and self-monitoring of blood glucose, (c) sends daily adherence assessment messages with weekly feedback and support, (d) sends messages with HIPAA-compliant access to A1c test results, and (e) provides access to a helpline tethered to a clinical pharmacist. In addition to the REACH elements, FAMS users received phone coaching and had the option to invite a support person (SP) to receive messages. FAMS also replaced REACH's general diet and exercise messages with messages tailored to users' coaching goal.

Text messages were tailored and sent by MEMOTEXT®, an algorithmic communications and data management platform supporting personalized user outputs and inputs via text messaging. Interventions using this platform have >90% retention rate [48]. We worked with MEMOTEXT to design FAMS functionality and conduct three rounds of internal beta testing during which research team members served as pseudousers to identify technical bugs and improve the user experience before usability and feasibility testing. Costs owed to MEMOTEXT included the initial development, a monthly maintenance fee of $200, and ~$0.07 per message sent (per-message costs decrease when more messages are sent).

### 3.1. Study Design

We conducted three iterative rounds of testing, each with a new sample of users. Each round included a single phone coaching session with the PP followed by two weeks of text message support for the PP and (if enrolled) the SP. PPs were adults with T2D recruited from three FQHCs in Nashville, TN, via flyers, interest cards, and referrals from clinic staff. Eligible PPs were receiving care at one of the clinic sites and taking diabetes medications (not caregiver administered). Both PPs and SPs had to be adults (≥18 years old), speak and read English, have a cell phone with text messaging, and provide a social security number (to process compensation). PPs and SPs were excluded if they had an existing diagnosis of dementia, were unable to orally communicate, or had an auditory limitation (for interviews). SPs verbally confirmed they could receive text messages. During enrollment, PPs were screened to confirm they could receive/read/send a message [47]. We aimed to enroll *n* = 6 PPs per round to satisfy the recommendation of three small studies with five participants in each round to identify all usability problems [49].

#### 3.1.1. Procedures

Study procedures were approved by the Vanderbilt University IRB. Trained research assistants (RAs) conducted eligibility screening and administered informed consent and survey measures in a private room at the PP's clinic. After enrollment, coaches (authors LSM and KJH) called the PP to complete phone coaching. Coaches then called and invited the SP to participate, asking if they would like to “receive text messages related to being a continued support” for the PP and complete an interview telling the research team what they thought about FAMS and how to improve it. Interested SPs gave their consent via phone and provided a preferred time of day to receive text messages.

Data from PPs' enrollment survey, coaching, and enrolled SPs were entered into REDCap™ and transferred automatically to MEMOTEXT via an application-programming interface for the tailoring and delivery of text messages. Compensation was $54 for PPs and $40 for SPs. The study did not pay for or supply cell phones or plans. Authors LSM and KJH met between rounds to discuss successes and shortcomings of the coaching protocols and SP enrollment process. Following each round of testing, we compiled PP- and SP-user feedback to improve FAMS and research processes. We received IRB approval before recruiting participants for the next round of testing.

#### 3.1.2. Measures

During the enrollment survey, PPs self-reported demographic and diabetes information and completed validated survey instruments to characterize the sample (health literacy [50], family involvement [16], and adherence to self-care behaviors [51, 52]). We also asked PPs if they were comfortable using their cell phone and text messaging. Glycemic control (A1c) was assessed via lab test with a blood sample drawn at enrollment.

Immediately after each coaching session, coaches recorded the PP's goal and type of family action—helpful or harmful—identified and addressed with psychoeducation and assessed the success of each phase of the coaching protocol using a “Coaching Assessment” we developed. Technical data collected by MEMOTEXT captured user engagement: we assessed PPs engagement as the number of text responses received out of the number of two-way texts sent. The FAMS user experience was assessed via semistructured interviews with the PP and, separately, the SP. Interviews were conducted by phone and included open-ended questions and closed- and open-ended question pairs (e.g., yes/no followed by “Why/Why not?” and 10-point scales followed by “Can you tell me why you chose that number?”). We asked participants to rate different aspects of the intervention (e.g., how easy the text messages were to understand, how helpful they were, and how much each FAMS component motivated them to reach their goal) on a scale from 1 (least favorable) to 10 (most favorable).

#### 3.1.3. Analysis

Interviews with PPs and SPs were audiorecorded with transcribed verbatim by an independent professional transcriber. Interview questions and responses were entered into REDCap. We exported survey and quantitative interview data from REDCap and conducted all descriptive analyses using Stata v. 13.1. Qualitative interview data were exported to Excel. Author LSM categorized and summarized feedback by FAMS intervention component.

### 3.2. Findings and Changes between Testing Rounds

We enrolled 19 PPs (mean per round *n* = 6.3). PP characteristics are shown in Table 2. Nine PPs invited a SP and seven SPs enrolled, including four boyfriends/girlfriends/fiancés, one spouse, one ex-spouse, and one son. There were no differences (significant or meaningful nonsignificant) between participants who chose to invite a SP and those who did not in any variable in Table 2. Of the 19 enrolled PPs, 17 (89%) completed an exit interview. Of the seven enrolled SPs, six (86%) completed an exit interview. Findings are described below with corresponding participant quotes in text and additional quotes in Table 3. Changes made between rounds and prior to a longer trial are described below and in Table 4.

#### 3.2.1. Phone Coaching

Table 5 shows Coaching Assessment data. Most participants focused on a helpful family action during skill building (examples in Table 5). All but one of the PPs who completed skill building were very confident (≥7 on a 10-point scale) they could apply the skill with an identified family member and, separately, doing so would result in a desired change (Table 5). In interviews, PPs said coaching helped them set a goal that was important to them (8.8 ± 1.4, range 6–10, example goals in Table 5). Several explained the coach helped them learn how to set a realistic and attainable goal:

*Oh, I thought [coaching] was helpful because it pinpointed what I could do and then helped me keep it doable or obtainable. It helped me focus on what I want to do, made me vocalize what I want to do, and then the coach part helped me make it - choose something that was obtainable. Also with the help, it was explained to me that it didn't need to be 15 solid minutes [of exercise]. I could do 5 minutes 3 times a day. And so, that really helped. So, it made me take a break at work, go walk in the mall for my 15 today. And tomorrow, take a break at work, or I could walk an entire floor, which would take about 5 minutes. You know, and then I'd do that 3 times and I'd have my 15 minutes. So, I could come home and watch a show and stand up and move around all the time it was on. Hey, it was movement and it's a start.* (PP, 50-year-old male, race unknown)

Two-thirds (67%) said coaching improved communication about diabetes with family members:

*I talk to my husband, and I explain about the goal, and then he helped me more. Like, now he probably is going to understand when I tell him, if we go out, what kind of food to order instead [of our regular order], because I already talked to him about what I talked about [in coaching].* (PP, 36-year-old Hispanic female)

Participants said they would want coaching once (33%) or twice (33%) per month in a longer intervention, and 65% said each session should last 15–30 minutes.

However, more than one in three participants (37%) did not “buy into” the coaching session (Table 5), and these same participants rated the coaching as less helpful in interviews. The most common reason (*n* = 4; 21%) was that the participant did not accept a connection between his/her goal and the actions of close loved ones. Coaches reported they had an insufficient amount of time to discuss family actions before asking PPs to think of how they would want such actions to change. Two PPs did not “buy into” the coaching session because the skill in the coaching protocol was not a good fit for the identified family actions or his/her family relationships (Table 5). For example, one participant had unsuccessfully tried assertive communication with his wife about the type of food she cooked and felt doing so again would not be productive (in his interview, this PP explained, “Because my family members, they ain't going to do that for me.” 68-year-old AA male). Finally, coaches reported spending a disproportionate amount of time on goal setting and rushing through the family-focused portions of the coaching protocol.

#### 3.2.2. Patient Participant Text Message Support

PPs rated the helpfulness of the goal support messages 8.4 ± 2.7 (range 1–10) and 80% said these messages motivated them to meet their goal. PPs liked that messages were short and easy to read and provided encouragement and support. One PP saved messages she wanted to memorize or apply to review in her free time. Three described using receipt of message as a cue to action to meet their goal and three others reported sharing messages with loved ones:

*You know, when I got the texts, I took a few minutes and did some arm rows and leg lifts and stretched my leg out, bring it to my stomach as much as I could.* (PP, 46-year-old Caucasian female)
*I mean, a lot of stuff I can explain to my wife that - she might understand it but seeing it for herself [in the messages], she understand it different.* (PP, 37-year-old AA male)

Two PPs and one SP wanted messages to be more interactive. One PP said texts that were not interactive make “people feel like they're being preached at.” Often, responses to one-way messages were received from PPs (e.g., “That's right!” and “I will, thank you”) and SPs (e.g., “We can work on it together”). Others appreciated not having to respond and said they were used to receiving text messages without responding.

PPs responded to 65% of the goal assessment messages and rated the helpfulness of these messages in keeping them on track with their goal 8.7 ± 2.1 (range 4–10). PPs appreciated getting feedback on their progress, and said the encouraging tone motivated them to keep trying to meet their goal:

*I would get a message: “Did you do it?” And then I said, “Oh, only 3 times.” And it said, “Ooh, You're halfway to your goal. What could - think about what would help you, you know, maybe get closer?” So, I didn't feel ashamed or I like “Ugh, I need to think of why didn't I do that?” You know? So, that part was really good about reaching my goal. It was very helpful - it reinforced the coaching that we did. It helped me not - I wanted to do better the second week. So, it's like, “Oh, dang it. I didn't do it the first week. I've got to do it this week.”* (PP, 50-year-old male, race unknown)

We made three changes to improve the goal assessment messages during testing (Table 4). First, we created functional flexibility to allow PPs to set a goal for ≥4 days. We altered feedback messages and functionality so a PP's response of 0–3 implied the participant had not met their goal, 4–6 implied success, and 7 received especially congratulatory feedback. Second, we widened the window during which MEMOTEXT could record a response. Third, we initially developed feedback to reference progress relative to the prior week (i.e., improvement, decline, or consistency) which resulted in nonresponse affecting feedback for the week in question and the subsequent week. Therefore, we changed the functionality of the feedback text to reference progress only when two weeks' responses were provided and to default to more basic feedback if not (e.g., “Keep trying to reach your goal each day. Use the tips you get in your texts. You can do better next week!”).

#### 3.2.3. Support Persons

Of the 19 PPs, nine (47%) invited a SP to enroll in the study and seven SPs were enrolled (we could not contact one; one declined). Six more PPs wanted to have a SP receive text messages but did not provide the SP's contact information to study staff. Reasons included the following: SP was ineligible (having no cell phone or being non-English-speaking), PP could not identify a SP, and SP expressed disinterest to the PP. Only four PPs (21%) did not want to invite a SP:

*Well, I guess I do have family members. But I just felt like this was my deal and I just wanted to own it myself because that really drives me more.* (PP, 50-year-old male, race unknown)
*My family members are really busy. I didn't want to put more weight on their shoulders.* (PP, 56-year-old Caucasian female)

Enrolled SPs reported the messages motivated them to talk with the PP about diabetes (9.3 ± 1.2, range 7–10) and improved their support of the PP (9.3 ± 1.2, range 7–10). When asked how many messages per week they would want to receive in a six-month intervention, SPs said three times per week (*n* = 3), daily (*n* = 2), or every other day (*n* = 1). Of the six SPs who completed an interview, four said FAMS increased their knowledge about diabetes and all reported discussing message content with PPs.

*I think it was a great experience for me because I had never known that much about diabetes and I was able to learn a great deal about it, you know, through those texts and then talking with [the PP]. I learned quite a bit…and in ways that I can actually help someone else. You know, if they're going through the same things…I also tried to, you know, watch what I eat as well. And, in turn, you know, we talk to each other about always trying to eat healthy and try to make the right choices and, you know, just try not to overdo things that would hurt her health-wise.* (SP, boyfriend)
*I think it was fun. I mean, I kind of looked forward to getting to see what it was going to be the next time, you know? [I would think], “Well, it's going to come through here in a minute.” You know, because it gave me an idea of what to - kind of get it in my head, you know, what I need to ask him for that evening, you know, just to throw it out there. I usually try to call him on my way home, and we discuss things. And that just kind of gives me an idea of something to actually throw out there, you know, and get his input on it. And he was always very open and honest and told me… I enjoyed the text messages.* (SP, ex-wife)

In round one, a SP-PP dyad reported the SP's text messages led to interpersonal conflict. The SP received this text message: “Do you argue with George about his diabetes? Take a step back. Next time ask what you can do to help George make better health choices” (name changed). The SP thought this message was sent to her because of something the PP told us:

*But there was one that kind of got me a little bit…it was kind of like I was being aggressive with him or something and that'd never been the case. And I was like, “I don't understand this one because I don't do that to him - pressure him about anything.” But anyway, that was the only one that was kind of strange…That one really needs to be worked on because that was disturbing (laughs). I mean, it just made me feel like I'd done something that maybe he had told you or something that I did.*

The PP was very upset about this:

*Y'all sent her text messages asking her why she want to argue with me when she's not arguing with me. She has never been negative about this; she's the one who wanted me to do this [study]. You all make her feel like I'm telling you bad shit about her and I'm not…That thoroughly pissed me off, and I texted back some pretty hot shit to them. I'm not sure if the computer understood it or not, but it made me feel better.*

We reported this unexpected adverse event to the IRB and fixed it for subsequent rounds. With family intervention experts, we reviewed and edited all SP messages to avoid insinuating SPs were performing a harmful action or experiencing conflict with the PP (Table 5). We also added this to the informed consent process for both the PP (i.e., conflict with friends/family members as a risk) and the SP (i.e., explained all of the SP text messages were sent randomly to all SPs in the study and not based on any information from PPs).

## 4. Lessons to Improve FAMS

In addition to described changes made between rounds of testing, we made several decisions and changes to FAMS for an upcoming evaluative trial based on these results (Table 4). Coaching sessions will occur monthly and are designed to last 20–30 minutes. The first session will allocate half of the session to assessing current diet or exercise activities and collaboratively setting a SMART goal, and the other half will be allocated to discussing the role of family actions/support in meeting and sustaining health goals. This session ends with a brief “Family Behavior Observation” skill building exercise, requesting that the PP observe the role of close loved ones as he/she tries to meet the goal for the first month. We hope this change will allow sufficient time for goal setting and increase the PP's acceptance of family members' role prior to asking PPs to identify desired family changes in subsequent sessions. We also created flexibility in the coaching protocol so coaches can tailor the skill to the PP* in vivo*. Each session involves the same elements (Table 1), but coaches choose the skill most applicable to PPs during sessions 2–5 (Table 4). We added the skill building exercise “Cognitive Behavioral Coping” for PPs who report not having supportive family members or experiencing persistent harmful family actions. We created a “wrap-up” session 6. We also made changes to our process for enrolling and inviting SPs to receive text messages (Table 4). With these changes, we hope PPs will understand the SP does not need to be a biological/legal family member and will have opportunity to ask the SP's permission to give study staff his/her contact information.

## 5. Conclusions

We designed FAMS to improve T2D adults' (a) adherence to diet and exercise recommendations and (b) self-efficacy and skills to engage loved ones in self-care goals in ways that facilitate behavior change. Through an iterative user-centered design process followed by rigorous and multimethod usability/feasibility testing [36], we designed an intervention that appeals to end users, is easy to use, and is applicable to a variety of patient and family situations. PPs said FAMS improved their diet/physical activity, communication with family members, and confidence in soliciting helpful actions/support. SPs reported FAMS increased the amount they communicated with PPs about diabetes and made it possible for them to be more helpful. Comments from users indicate families find it difficult to know how to communicate about diabetes or support diabetes self-care efforts and appreciated concrete suggestions provided during coaching or in text messages. Over half of the PPs identified a harmful family action, but fewer (13%) focused on the harmful action in skill building. Patients who discussed harmful actions often preferred either asking for a helpful action to replace or offset the harmful action or asking for a helpful action from another family member instead of dealing with the family member performing the harmful action. These are productive ways to manage harmful actions, as helpful actions have been found to protect against the detrimental effects of harmful actions on patients' A1c [12].

We discovered limitations of FAMS. For instance, only 37% of PPs successfully invited a SP and a few PPs did not want to discuss family issues at all. We hope to have developed appropriate and effective solutions to these problems, but the success of these changes remains unknown. We do know, however, that our iterative process dramatically reduced technical bugs and problems with content, functionality, and study processes, thereby maximizing our ability to evaluate FAMS. FAMS targets patients' adherence to diet and exercise, but other self-care behaviors (e.g., medication adherence, self-monitoring of blood glucose) are impacted by family actions [2, 12] and affect diabetes control [2, 12].

### 5.1. Future Research

Future work should assess patients' expectations of others' involvement in diabetes self-care. Such expectations may moderate the relationship between family involvement and patients' outcomes. For instance, in other disease contexts, helpful [53] and harmful [54] family involvement have been shown to affect dietary adherence [54], self-efficacy [53], and depressive symptoms [53] differently based on perceptions of the role of family in adults' self-management. Thus, future research should examine whether individuals with T2D who view their illness as an individual concern benefit from family involvement or interventions like FAMS. Additionally, we found SPs may react negatively to the idea that their actions might be less than helpful or they might nag or argue with the patient. Future intervention efforts may benefit from normalizing nagging and arguing and inadvertently making self-care more challenging. This may make it less emotionally fraught for families to identify and replace these actions with more helpful ones.

Although mHealth interventions have improved adherence and glycemic control among adults with T2D [55, 56], engaging family members in these interventions remains understudied. To our knowledge, the only other mHealth intervention involving a patient-selected family member/support person for adults with T2D is mHealth + CarePartner [57, 58]. Both mHealth + CarePartner and FAMS were designed to be inclusive of patients who live alone, recruit from community clinics, and provide tailored mobile communications to an adult family member/friend. However, there are key differences between Piette et al.'s [59] approach to engaging a support person and our own. CarePartners receive weekly information about the patients' health status along with tailored advice about how to help. In contrast, FAMS does not provide information about patients' progress to SPs but rather aims to empower the patient to identify and communicate support needs. Lessons learned from FAMS and mHealth + CarePartner will improve future efforts to involve meaningful others in adults' T2D self-management.

### 5.2. Strengths and Limitations of Our Approach

We enrolled a small convenience sample with no control group and users experienced the intervention for a brief time, limiting our understanding to participants' anecdotal accounts and short-term engagement data. “Down time” was required for troubleshooting technical bugs, making fixes/improvements, and adding new functionality. As a result, we had to wait between each development phase and paused recruitment between rounds of usability/feasibility testing. These necessary and inevitable occurrences—rarely accounted for in study planning or funding schedules—nearly doubled the length of FAMS development and testing. However, similar “down time” during an evaluative trial would have been much more problematic and costly. Because identifying and fixing bugs are inevitable with mHealth interventions, we stress the importance of starting rigorous multiround testing early in the design/development process rather than waiting until the intervention is developed and functioning properly to allow users to experience it. More time and energy in these phases likely pay off with faster evaluative trials with more engagement, less attrition, and fewer study limitations associated with functional problems [36]. Such work is especially critical when designing mHealth interventions for underserved and disadvantaged patients.

## Figures and Tables

**Table 1:** FAMS intervention components and description.

	Component	Description
Patient participants	Phone coaching	Each session includes the following: (i) Goal setting: identify a SMART (specific, measurable, attainable, relevant, time-bound) diet or exercise goal (ii) Discussing helpful & harmful family actions: identify and differentiate between family members' helpful and harmful actions related to the SMART goal (iii) Skill building: complete exercises in relation to a specific wanted helpful action or unwanted harmful action and confirm comprehension via role playing or teaching back (iv) Verbal contract: make a plan to employ the skill with a specific family member and assess participant's self-efficacy to do so

Patient participants	Text messages	Goal support messages: one-way texts with tips and motivational content tailored to the SMART goal set during phone coaching: (i) 2 texts each week are not tailored to goal type *Changing your lifestyle isn't easy, but small steps matter! Reaching your SMART goal today can add up to big improvements!* (ii) 1 text each week is tailored to the participant's goal type (diet or exercise) *Many people forget about their diet goals around holidays or vacations. Plan ahead and prepare for these changes in your daily routine.* *Work your SMART exercise goal into your day. Exercise while doing other things like talking on the phone or watching TV.* The one-way text is sent within users' preferred window of time (the exact time varies within this window)
		Goal assessment messages: Each Saturday, participants receive a two-way/interactive text tailored to their goal *Your goal was to walk 15 minutes each day. How many days did you meet this goal last week (Sun-Sat)? Reply with the number of days, 0–7.* (i) Participant's response is followed by a feedback text, tailored to the response *Excellent progress! Consider what motivated you to do better this week than in weeks before. Keep up your good work!*

Support persons	Invitation	Participants can choose to invite an adult family member or friend to receive text messages (i) Participants who invite a support person provide his/her first name and phone number (ii) Support person must be English-speaking and own a cell phone (iii) Study staff calls the support person to invite him/her to participate, complete informed consent, enroll via phone

Support persons	Text messages	Each week, enrolled support persons receive 3 one-way texts with content tailored to the patient participant's selected goal, name, gender *Bob has set a goal to improve his diet. Ask him about it the next time you speak to him.* (i) 2 texts each week are not tailored to goal type *Often we want to help our loved ones with their health but don't know how. Ask Bob what you can do to make it easier for him to reach his goal.* (ii) 1 text each week is tailored to the patient participant's goal type (diet or exercise) *Be sensitive about eating treats in front of Bob as it may tempt or make him feel left out. Instead, choose treats you can enjoy together.* *Dancing is the most fun exercise you can do at home. Help Bob meet his exercise goal by putting on his favorite jam and starting a 2 person party!* The one-way texts are sent at the support person's preferred time of day

**Table 2:** Participant characteristics.

M ± SD or *n* (%)	Total *N* = 19	Support Person Invited	
		Yes *n* = 9	No *n* = 10
*Demographics*			
Age, years	51.7 ± 10.2	52.0 ± 9.4	51.3 ± 11.6
Gender, female	10 (53)	4 (44)	6 (60)
Race^a^			
Caucasian/white	7 (39)	4 (44)	3 (33)
African American/black	8 (44)	4 (44)	4 (44)
Others^b^	3 (17)	1 (11)	2 (22)
Education, years	12.8 ± 2.5	13.4 ± 1.9	12.2 ± 2.9
Annual household income, US$^a^			
<10,000	8 (44)	3 (33)	5 (50)
10,000–34,999	7 (39)	3 (33)	4 (40)
≥35,000	3 (17)	3 (33)	0 (0)
Limited health literacy (BHLS)	2 (11)	1 (11)	1 (10)
*Family characteristics*			
Married/partnered	5 (26)	3 (33)	2 (20)
Helpful actions (DFBC-II)	1.6 ± 0.7	1.5 ± 0.8	1.6 ± 0.7
Harmful actions (DFBC-II)	1.4 ± 0.7	1.2 ± 0.7	1.6 ± 0.8
*Diabetes characteristics*			
Diabetes duration, years	6.9 ± 5.9	5.1 ± 5.1	8.3 ± 6.4
Insulin status, taking insulin	8 (42)	4 (44)	4 (40)
*Previous cell phone use*			
Comfortable using cell phone	18 (95)	9 (100)	9 (90)
Used text messages	17 (90)	8 (89)	9 (90)
*Self-care adherence*			
Medication adherence (ARMS-D)	25.8 ± 2.8	25.9 ± 3.9	25.7 ± 1.4
General diet (SDSCA)	3.6 ± 2.5	3.7 ± 2.8	3.5 ± 2.4
Specific diet (SDSCA)	3.5 ± 1.7	3.3 ± 1.6	3.6 ± 2.0
Exercise (SDSCA)	2.0 ± 1.5	2.2 ± 1.6	2.1 ± 1.5
SMBG (SDSCA)	3.9 ± 3.1	3.0 ± 3.2	4.7 ± 2.9
*Glycemic control* (A1c, %)	7.4 ± 1.6	7.4 ± 1.6	7.5 ± 1.7

^a^One participant refused/did not know.

^b^Two Hispanic people and one Native American.

*Note.* Mann–Whitney *U* and Fisher's exact tests identified no association between any variable and inviting a support person. A1c, hemoglobin A1c; BHLS, Brief Health Literacy Screen (limited if score ≤ 9); ARMS-D, Adherence to Refills and Medications Scale for Diabetes medication taking subscale (possible range 7–28; higher scores indicate more adherence); DFBC-II, Diabetes Family Behavior Checklist-II (possible range 1 = never to 5 = once a day); SDSCA, Summary of Diabetes Self-Care Activities (possible range 0–7; it indicates number of days adherent per week); and SMBG, self-monitoring of blood glucose.

**Table 3:** Participant feedback on FAMS.

*Phone coaching *	

*I would have never set a goal [without the coaching]. I would just, you know - just took it day-by-day, not really set a goal. *(PP, 57-year-old AA female)	

*Well, it gave me incentive to try to cut down - my goal was to cut down on my colas. And you gave me incentive to do that, but you also left me some leeway, you know, where I could still maybe have one a day, you know. But you gave me some wiggle room instead of just saying, “Stop it.” You know, it was almost like a detox, a gradual detox from cola.* (PP, 60-year-old Caucasian male)	

*It made me be more honest about my health issues to my fiancé. *(PP, 46-year-old Caucasian female)	

*Well, it made me want to tell [my family] more about [my diabetes]. And then it makes them want to be more interested, you know, in what I have to say. If they come to realize how important, you know, diabetes is. *(PP, 55-year-old AA female)	

*Patient participant text message support*	

*I liked reading them. They were short, so it was not like I spent a lot of time reading them…It felt like I had a little bit of support that normally you just don't have. You had backup that you just normally - like I say, I have nobody, and it felt like I'd come to have somebody there for a while.* (PP, 36-year-old Hispanic female)	

*Well, when I get a text from the program - then people are like, “Who's that?” I'm like, “Oh, it's the study I'm doing for my diabetes.” And they're like, “Oh. Well, what does it do?” You know, so, it opens a conversation for some people, you know, that I wouldn't have told probably.* (PP, 50-year-old male, race unknown)	

*And it wasn't real intrusive. Just a gentle reminder, “Hey,” you know, a text popped up. And just addressed some different things I might possibly be going through. And, you know, it was perfect. *(PP 58-year-old Caucasian male)	

*Support person text messages*	

*I think it was fun. I mean, I kind of looked forward to getting to see what it was going to be the next time, you know? [I would think], “Well, it's going to come through here in a minute.” You know, because it gave me an idea of what to - kind of get it in my head, you know, what I need to ask him for that evening, you know, just to throw it out there. I usually try to call him on my way home, and we discuss things. And that just kind of gives me an idea of something to actually throw out there, you know, and get his input on it. And he was always very open and honest and told me… I enjoyed the text messages. *(SP, ex-wife)	

*Interviewer: Did you discuss any of the text messages with [the PP]?* *Every last one of them. *(SP, husband)	

*It made us talk when I got a text and she got a text…She gets her text. I get my text, we sit and discuss them when we both get them. Because I get mine early in the morning, and she gets hers at night. It reminds [family members] of what they're not doing, or it brings it up so me and her can talk about it. *(SP, boyfriend)	

*Overall opinions and experiences*	

*It reminds her that somebody actually cares about what she's doing, what's going on with her and her health. *(SP; fiancé)	

*It was a really good goal for me. And to do it, it's been wonderful. [My husband] and I have been doing stuff more together. And I've been getting more exercise because of it. And, you know, it's had the benefit of we're more tired by the end of the day. *(PP, 61-year-old Caucasian female)	

AA, African American; PP, patient participant; and SP, support person.

**Table 4:** FAMS intervention changes.

	Component	Usability & feasibility testing	Randomized controlled trial
Patient participants	Phone coaching	(i) Skill building exercise and type of family action assigned by round of usability testing (a) Assertive communication to ask for a needed helpful action (Round 1) (b) Assertive communication to stop/redirect an unwanted harmful action (Round 2) (c) Problem solving to identify how family members can help overcome a barrier to goal completion (Round 3)	(i) Session 1 focuses on collaborative goal setting and brief skill building exercise (Family Behavior Observation) with homework to observe family actions (ii) Coach and participant collaboratively chose whether to focus on a helpful or harmful family action for skill building (iii) Coach chooses skill building exercise based on participant needs during sessions 2–5 (a) Assertive communication (b) Problem solving (c) Cognitive behavioral coping

Patient participants	Text messages	*Content*	
		(i) Goal assessment message and feedback assume a daily goal *Your goal was to walk 15 minutes each day. How many days did you meet this goal last week (Sun-Sat)? Reply with the number of days, 0–7.* (a) Feedback based on number of days goal was completed out of 7 days; success was identified as meeting goal 7 days	(i) Goal assessment message and feedback accommodate a goal for ≥4 days per week *Your goal was to walk 15 minutes 4 days. How many days did you meet this goal last week (Sun-Sat)? Reply with the number of days, 0–7.* (a) Feedback congratulated respondent for meeting the goal, 4–6 days, and challenged him/her to strive for 7 days next week
		*Functionality*	
		(i) Participants have 2 hours to respond to goal assessment text (a) Feedback is tailored to participants' response from the week in question and the prior week (b) Nonresponse affects feedback for the week in question and the next week	(i) Participants have 4 hours to respond to goal assessment text (a) Feedback is tailored to participants' response from the week in question and if the participant responded the prior week, whether goal attainment improved/declined/was consistent (b) Nonresponse only affects feedback for the week in question

Support persons	Invitation & enrollment	(i) Participant given the option to invite a “family member” upon enrollment (by enrolling research assistant), only once	(i) Participant given option to invite a “support person” (SP) at end of each coaching session (by coach), multiple times (a) Emphasized to participant that all SP involvement occurs via phone (ii) Told SP all text messages are sent to all SPs in the study and are not selected based on anything the PP reported about the SP or their relationship

Support persons	Text messages	*Content*	
		(i) Some texts used language like “nagging” and “diabetes cop” *Do you examine Beth's plate and nag them during meals? Think of more helpful ways to promote better eating besides being a diabetes cop.* *Do you argue with Beth about her diabetes? Take a step back. Next time ask what you can do to help Beth make better health choices.* *Your role as a loved one is to support Beth in meeting her health goal by asking how you can be helpful. Remember not to be the diabetes police!*	(i) Avoided language that can be perceived as accusatory or offensive *Instead of asking Beth what she is doing to take care of her diabetes, ask what you can do to help.* *Even if you disagree with Beth about her health, be gentle and find a way to help her reach her goals.* *Support Beth by brainstorming together ways you can help her be successful in meeting her health goals.*
		*Functionality*	
		(i) Daily texts	(i) Three texts per week

**Table 5:** Coaching Assessment data evaluating FAMS protocol success.

*Goal setting*		
Was the patient able to set a SMART goal?		
47%: yes, independently		
32%: yes, with help from the coach		
21%: no, needed the coach to set a goal for them		
What type of goal was set?		
53%: diet (e.g., decrease to one 12-ounce soda, eat 3 servings of vegetables)		
47%: exercise (e.g., walk 15 min or until feet hurt, lift canned goods as weights for 10 min)		

*Discussing helpful & harmful family actions*		
Could the patient identify helpful family actions he/she had experienced?		
74%: yes, independently		
16%: yes, with help from the coach		
10%: no		
Could the patient identify harmful family actions he/she had experienced?		
42%: yes, independently		
16%: yes, with help from the coach		
42%: no		

*Skill building*		
84% engaged in the skill building exercise (percentages below reflect the 16 participants who completed skill building)		
Desired change used for skill building:		
86%: wanted helpful action (e.g., choose healthy places to eat out, cook meals with me, exercise with me, do accountability calls or texts)		
13%: unwanted harmful action (e.g., stop bringing unhealthy food to my house, stop bringing food over after dinner time)		
68% were able to role play or teach back the skills learned		
Was there any portion of the coaching protocol the patient did not “buy into”?		
21% (*n* = 4) did not accept a connection between health goal and loved ones' actions		
5% (*n* = 1) tried assertive communication with his wife with no results—the assertive communication skill was not a good fit		
5% (*n* = 1) has good support and lacks personal motivation		
5% (*n* = 1) didn't accept idea that family could be harmful to diabetes self-care		

*Verbal contract*		
79% made a verbal contract to implement the skill with an identified family member		
Participants' confidence he/she can complete the verbal contract on scale 1–10 (*How confident are you that you can use assertive communication to ask your sister to walk with you?*)		
1 – 6% (not at all confident)	8 – 18%	10 – 53% (totally confident)
7 – 12%	9 – 12%	
Participants' confidence in success on scale 1–10 (*How confident are you that doing so will result in your sister walking with you?*)		
1 – 11% (not at all confident)	8 – 17%	10 – 50% (totally confident)
7 – 11%	9 – 11%	
